# STAT-1 Knockout Mice as a Model for Wild-Type Sudan Virus (SUDV)

**DOI:** 10.3390/v13071388

**Published:** 2021-07-17

**Authors:** Olivier Escaffre, Terry L. Juelich, Natasha Neef, Shane Massey, Jeanon Smith, Trevor Brasel, Jennifer K. Smith, Birte Kalveram, Lihong Zhang, David Perez, Tetsuro Ikegami, Alexander N. Freiberg, Jason E. Comer

**Affiliations:** 1 Department of Pathology, University of Texas Medical Branch at Galveston, Galveston, TX 77555, USA; olescaff@UTMB.EDU (O.E.); tljuelic@UTMB.EDU (T.L.J.); jeksmith@UTMB.EDU (J.K.S.); bkkalver@UTMB.EDU (B.K.); lihzhang@UTMB.EDU (L.Z.); teikegam@utmb.edu (T.I.); 2 XTR Toxicologic Pathology Services LLC, Sterling, VA 20165, USA; natasha.neef@gdneef.plus.com; 3 Office of Regulated Nonclinical Studies, University of Texas Medical Branch at Galveston, Galveston, TX 77555, USA; chmassey@UTMB.EDU (S.M.); jensmit1@UTMB.EDU (J.S.); trbrasel@UTMB.EDU (T.B.); 4 Department of Microbiology and Immunology, University of Texas Medical Branch at Galveston, Galveston, TX 77555, USA; 5 The Center for Biodefense and Emerging Infectious Diseases, University of Texas Medical Branch at Galveston, Galveston, TX 77555, USA; 6 Texas A&M University Division of Research, Texas A&M University, College Station, TX 77843, USA; dadperez@tamu.edu; 7 Sealy Institute for Vaccine Sciences, University of Texas Medical Branch at Galveston, Galveston, TX 77555, USA; 8 Institute for Human Infections and Immunity, University of Texas Medical Branch at Galveston, Galveston, TX 77555, USA; 9 Institute of Translational Sciences, University of Texas Medical Branch at Galveston, Galveston, TX 77555, USA

**Keywords:** ebolavirus, filovirus, SUDV, STAT-1 knockout mice, animal model

## Abstract

Currently there is no FDA-licensed vaccine or therapeutic against Sudan ebolavirus (SUDV) infections. The largest ever reported 2014–2016 West Africa outbreak, as well as the 2021 outbreak in the Democratic Republic of Congo, highlight the critical need for countermeasures against filovirus infections. A well-characterized small animal model that is susceptible to wild-type filoviruses would greatly add to the screening of antivirals and vaccines. Here, we infected signal transducer and activator of transcription-1 knock out (STAT-1 KO) mice with five different wildtype filoviruses to determine susceptibility. SUDV and Marburg virus (MARV) were the most virulent, and caused 100% or 80% lethality, respectively. Zaire ebolavirus (EBOV), Bundibugyo ebolavirus (BDBV), and Taï Forest ebolavirus (TAFV) caused 40%, 20%, and no mortality, respectively. Further characterization of SUDV in STAT-1 KO mice demonstrated lethality down to 3.1 × 10^1^ pfu. Viral genomic material was detectable in serum as early as 1 to 2 days post-challenge. The onset of viremia was closely followed by significant changes in total white blood cells and proportion of neutrophils and lymphocytes, as well as by an influx of neutrophils in the liver and spleen. Concomitant significant fluctuations in blood glucose, albumin, globulin, and alanine aminotransferase were also noted, altogether consistent with other models of filovirus infection. Finally, favipiravir treatment fully protected STAT-1 KO mice from lethal SUDV challenge, suggesting that this may be an appropriate small animal model to screen anti-SUDV countermeasures.

## 1. Introduction

The family *Filoviridae* includes *Cuevavirus*, *Ebolavirus*, and *Marburgvirus,* but only in the two latter genera can viruses pathogenic to humans be found [1,2,3]. Specifically, Zaire ebolavirus (EBOV), Sudan ebolavirus (SUDV), Bundibugyo ebolavirus (BDBV), Taï Forest ebolavirus (TAFV), Marburg virus (MARV), and Ravn virus (RAVV) have been responsible for multiple outbreaks of hemorrhagic fever throughout Africa [2,3]. EBOV, SUDV, and MARV cause case fatality rates (CFR) ranging from 25 to 100%, and typically larger outbreaks than BDBV and RAVV, whose CFR can range from 25 to 80% [2,4,5,6,7,8,9,10,11]. Only one case of TAFV infection was reported in 1994, and the patient survived [2]. Virus transmission between humans occurs by contact of infectious bodily fluids on skin abrasions or mucosal tissues, including during sexual intercourse [12,13]. Following the incubation period, patients experience an influenza-like syndrome that includes headache, fever, arthralgia, myalgia, gastro-intestinal symptoms, chest pain, and sore throat. The disease then progresses into hemorrhagic complications such as maculo-papular rash, petechiae, conjunctival hemorrhage, epistaxis, hematemesis, internal bleeding, and shock [14].

SUDV infection in non-human primates (NHP) closely resembles the disease seen in human patients. This recapitulation of human disease established the NHP model as the gold standard model for research purposes. However, these models are often not suitable during the initial screening and testing steps of vaccine and therapeutic candidates, due to ethical reasons and cost [15].

Immunocompetent mice or other rodents such as guinea pigs and hamsters are resistant to wild-type filovirus infection, resulting in no or, at best, minimal clinical disease [16]. To circumvent this issue, rodent-adapted viruses have been generated by serial passages in either suckling mice, guinea pigs, or hamsters. This has introduced virus genome mutations that allows antagonization of interferon response [17,18] and disease in these otherwise resistant models [17,19,20,21,22,23,24,25,26,27]. However, rodent-adapted strains, as opposed to wild-type virus isolates from human patients, may contain changes in viral proteins that are often targets for vaccines and therapeutics, including those involved in initial attachment and cell entry. In response to this, immunocompromised rodent models that lack one or more components of the immune response have been developed to study wild-type filovirus disease [19,28,29,30,31,32,33,34,35,36,37,38,39] and are used as platforms to evaluate anti-filovirus therapeutic and vaccine candidates [26,28,29,30,31,38,39,40,41,42,43,44,45,46,47]. Specifically, these models have included the interferon-α/β receptor knockout (IFNAR KO), the double interferon-α/β and γ receptor (IFNAGR) KO, interferon-γ receptor (IFNGR) KO, the cytoplasmic signal transducer and activator of transcription-1 protein (STAT-1) KO, the severe combined immunodeficiency (SCID) KO mice, and STAT-2 KO hamsters. The virulence of a given virus species or strain can vary across these different models, which have a distinct inability to mount a type I or type I + II IFN response or an adaptive immune response to combat the infection. This variability in virulence has been observed using the same mouse model on the same or different genetic backgrounds between different studies, causing difficulties in interpreting and comparing data [28,29,30,32,37,38,39,47,48]. Furthermore, histopathological data in filovirus-challenged IFNAR and STAT-1 KO mice are inconsistent or lacking [29,35,36,37,38,47]. Taken together, there is still a need for further characterization of EBOV, MARV, and other filoviruses in these immunocompromised mouse models. This will be critical to allow for comparability of virulence, pathogenicity, and transmission between virus species, as well as to determine which model (s) is ideal for initial testing of filovirus countermeasures.

As part of the NIH/NIAID Animal Models of Infectious Diseases Contract, we further characterized the virulence and pathogenicity of wild-type filoviruses in mice lacking the cytoplasmic signal transducer and activator of transcription-1 protein (STAT-1 KO).

## 2. Materials and Methods

### 2.1. Ethics Statement

This study complied with the Final Rules of the Animal Welfare Act regulations (9 CFR Parts 1, 2, and 3) and Guide for the Care and Use of Laboratory Animals: Eighth Edition (Institute of Laboratory Animal Resources, National Academies Press, 2011; the Guide). This study was conducted in UTMB’s AAALAC (Association for the Assessment and Accreditation of Laboratory Animal Care)-accredited facility and was approved by UTMB’s Institutional Animal Care and Use Committee (protocol number 1712017, approved 1 December 2017).

### 2.2. Cells and Viruses

Marburg virus (MARV) Angola, BEI Resources NR-48866; Ebola virus (EBOV) Zaire Kikwit, BEI Resources NR-48867; Sudan virus (SUDV) Gulu, BEI Resources NR-48868; Bundibugyo virus (BDBV), BEI Resources NR-48869; and Taï Forest virus (TAFV), BEI Resources NR-48870 were confirmed sterile including absence of mycoplasma and endotoxin. All stocks were subjected to deep sequencing analysis with >99% homology to reference sequences. Virus titers were determined as previously described [49].

### 2.3. Mouse Model

Male and female 7–11-week-old STAT-1 knockout on a 129S6 background (Taconic, Rensselaer, NY, USA).

### 2.4. Mouse Challenges and Observations (Filovirus Susceptibility Study)

Five groups of 5 mice (2 male and 3 female or 3 male and 2 female) were challenged with 1.0 × 10^2^ pfu of either MARV, EBOV, SUDV, BDBV, or TAFV by i.p. injection. Virus challenge day was defined as Day 0. Virus stocks were diluted in basal minimum essential medium (MEM) to the targeted challenge dose per 100 μL. The viral dose was verified by standard plaque assay [49]. Mice were weighed daily through day 9 post-challenge then every 3 to 4 days until the end of the study on day 21. All surviving animals were scheduled to be humanely euthanized on that day. Animals were monitored daily by visual examination and scored according to the following criteria: Score of 1: healthy. Score of 2: displaying mild signs of lethargy, fur ruffling. Score of 3: Score 2 + hunched posture. Score of 4: Score 3 + limited mobility. Score of 5: Score 4 + inability to reach food or water, OR a >20% weight loss; any score of 5 required immediate euthanasia.

### 2.5. Mouse Challenge and Observations (SUDV Serial Dosing Study)

Five groups of 5 mice (2 male and 3 female or 3 male and 2 female) were challenged with SUDV at a target dose of either 10^0^, 1.0 × 10^1^, 1.0 × 10^2^, 1.0 × 10^3^, or 1.0 × 10^4^ pfu per animal by i.p. injection. Viral dose determination, body weight measurements, and clinical scoring were performed as described previously for the susceptibility study. End of study was defined as 14 days post-challenge.

### 2.6. Mouse Challenge and Observations (Natural History Study)

Forty-six mice (23 male and 23 female) were challenged with SUDV at a target dose of 1.0 × 10^2^ pfu per animal by i.p. route on day 0. No back titration of the actual delivered dose was performed. A separate group of 7 mice (3 male and 4 female) were euthanized as naïve controls the same day. Seven pre-selected SUDV-infected mice (3 male 4 female or 4 male and 3 female) were scheduled to be euthanized daily from day 0 through day 6, with the remaining animals serving as replacements for animals that succumbed to infection prior to scheduled euthanasia time points. Mice were weighed daily until the end of the study on day 7. For this experiment, clinical scoring was refined slightly to the following criteria: Score of 1: healthy. Score of 2: ruffled and lethargic. Score of 3: score of 2 + hunched posture and orbital tightening. Score of 4: score of 3 + reluctance to move when stimulated or ≥20% body weight loss, and required immediate euthanasia. 

### 2.7. Mouse Challenge and Observations (Favipiravir Efficacy Study)

Groups of 6 mice (3 male and 3 female) were challenged with 1.0 × 10^2^ pfu of SUDV via i.p. injection. No back titration of the actual delivered dose was performed. Starting 1-hour post-challenge, mice were treated with 150 mg/kg of Favipiravir (AdooQ Bioscience, Irvine, CA, USA) in 0.5% methylcellulose (100 µL volume) twice daily for 8 days via oral gavage. Animals were monitored for 21 days and scored as described for the natural history study.

### 2.8. SUDV qRT-PCR

50 µL of sera was added to 250 µL of TRIzol LS (Invitrogen, Carlsbad CA, USA) and stored at ≤−70 °C until RNA extraction using a Zymo Research Direct-zol™ RNA MiniPrep kit (Zymo Research, Irvine CA, USA). Final elutions (containing purified RNA) were stored at ≤−70 °C until use. For sample quantification, each assay plate included a standard curve prepared using an HPLC-purified synthetic EBOV RNA standard containing the conserved EBOV glycoprotein sequence (GenBank accession no. AY729654) ranging from 1.0 × 10^3^ to 1.0 × 10^10^ genome equivalents/µL (GEq/μL]. Samples were run in duplicate wells. For the RT-PCR, QuantiFast Probe RT-PCR Master Mix (Qiagen, Germantown MD, USA), and QuantiFast RT Mix (Qiagen, Germantown MD, USA) were used in conjunction with Forward primer: 5′- ggA Tgg AgC TTT CTT CCT CTA Tg -3′, Reverse primer: 5′- TAC CCC CTC AgC AAA ATT gAC T -3′, and Probe: 5′-6FAM- CAg gCT ggC TTC AAC TgT AAT TTA CAg Agg -MGBNFQ-3′. The RT-PCR was performed using a Bio-Rad CFX96TM Real-Time PCR detection system.

### 2.9. Hematology and Serum Chemistry

Blood was collected in EDTA tubes to analyze common hematology parameters using a HEMAVET 950 Multispecies Hematology Analyzer (Drew Scientific, Miami Lakes, FL, USA). Measured parameters included total white blood cell counts (WBC), total and percent neutrophils (NE), lymphocytes (LY), monocytes (MO), eosinophils (EO), basophils (BA), and monocytes (MO). Additional blood volume was collected in SST tubes, processed to serum, and analyzed for changes in clinical chemistry using a VetScan VS2 Chemistry Analyzer and Comprehensive Diagnostic Profile rotors (Abaxis, Union City, CA, USA) as previously described [39]. Samples collected from uninfected animals euthanized on day 0 served as baseline.

### 2.10. Histology

Liver and spleen samples were harvested from mice at necropsy, fixed in 10% neutral-buffered formalin, and provided to Experimental Pathology Laboratories, Inc. (EPL^®^, Sterling VA, USA). Tissues were processed, paraffin embedded, sectioned at 4–6 µm (one spleen section and two liver sections/mouse), and stained via hematoxylin and eosin using common histology techniques. All tissue sections were examined by a board-certified pathologist.

## 3. Results 

### 3.1. Filovirus Susceptibility Study in STAT-1 KO Mice

**Figure 1:**
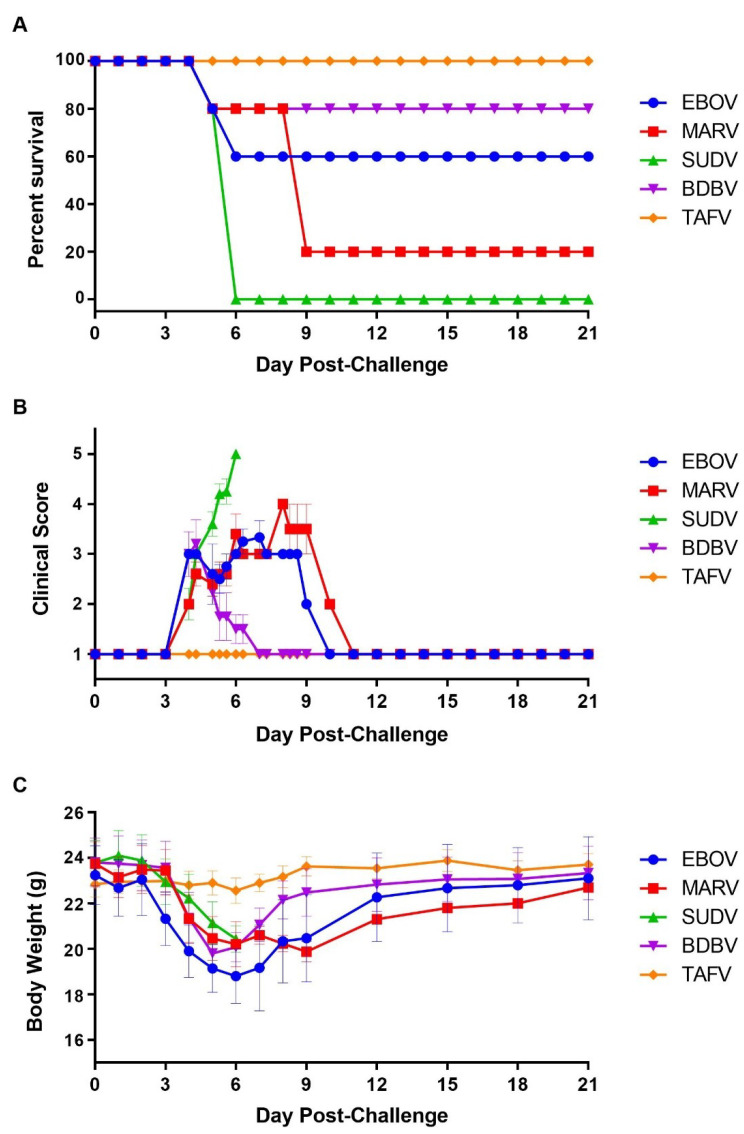
Screening of STAT-1 KO Mice for Susceptibility to Wild-type Filoviruses. Mice (N = 5) were challenged with a targeted dose of 1.0 × 10^4^ pfu and observed for up to 21 days for survival (**A**), clinical presentation of disease (**B**), and body weight loss (**C**). Symbols represent group mean clinical scores (**B**) or body weights (**C**); bars indicate standard error.

Results following challenge with 1.0 × 10^4^ pfu demonstrated that SUDV and MARV were the most virulent in the STAT-1 KO mice, causing 100% and 80% lethality, respectively (Figure 1A). SUDV-infected mice met euthanasia criteria on day 5 and 6, while most MARV-infected mice were euthanized by day 9. EBOV, BDBV, and TAFV challenge resulted in 40%, 20%, and no mortality, respectively. Two of the five EBOV-infected animals were euthanized on days 5 and 6 (n = 1 per day); one BDBV-challenged mouse was euthanized on day 4. Mice challenged with SUDV, MARV, EBOV, and BDBV exhibited clinical signs of disease and mean weight loss, starting between days 3 and 4, while all mice challenged with TAFV remained normal throughout the study (Figure 1B,C). Survivors initially infected with MARV, EBOV, and BDBV returned to baseline (clinically healthy) on days 11 (1 mouse), 10 (3 mice), or 7 (4 mice), respectively. All surviving mice returned to pre-challenge body weight by the end of the study (Figure 1B,C).

### 3.2. SUDV Serial Dosing Study in STAT-1 KO Mouse Model

**Figure 2:**
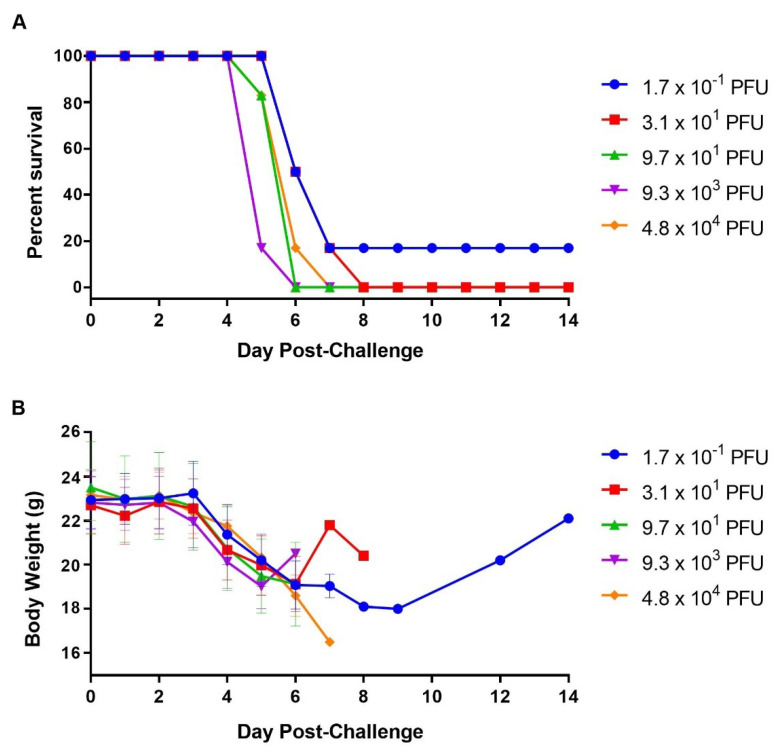
Dilutional Virulence of SUDV in STAT-1 KO mice. Mice (N = 5) were challenged with various doses of SUDV and observed for 14 days for survival (**A**), and body weight loss (**B**). Symbols represent group mean body weights (**B**); bars indicate standard error.

To assess the relationship between infective dose and virulence in the SUDV-challenged STAT-1 KO model, we infected five groups of mice (n = 5 per group) with a target of 1.0–1.0 × 10^4^ pfu per animal. SUDV was 100% lethal down to 3.1 × 10^1^ pfu (actual dose) with mice succumbing to infection between days 5 and 8 (Figure 2A). The lowest dose tested (1.7 × 10^−1^ pfu) still caused 83% mortality rate within the same time frame, with the one surviving mouse returning to normal and initial body weight on days 11 and 14, respectively (Figure 2B,C). Our results demonstrated that the virulence of SUDV in this animal model is dose dependent.

Increased clinical scores (data not shown) and mean body weight loss (Figure 2B) were observed in most groups following challenge. The increase in mean body weight in two groups at day 6 or 7 (Figure 2B) stemmed from lighter animals that succumbed to disease prior to these time points.

### 3.3. SUDV Natural History Study

**Figure 3:**
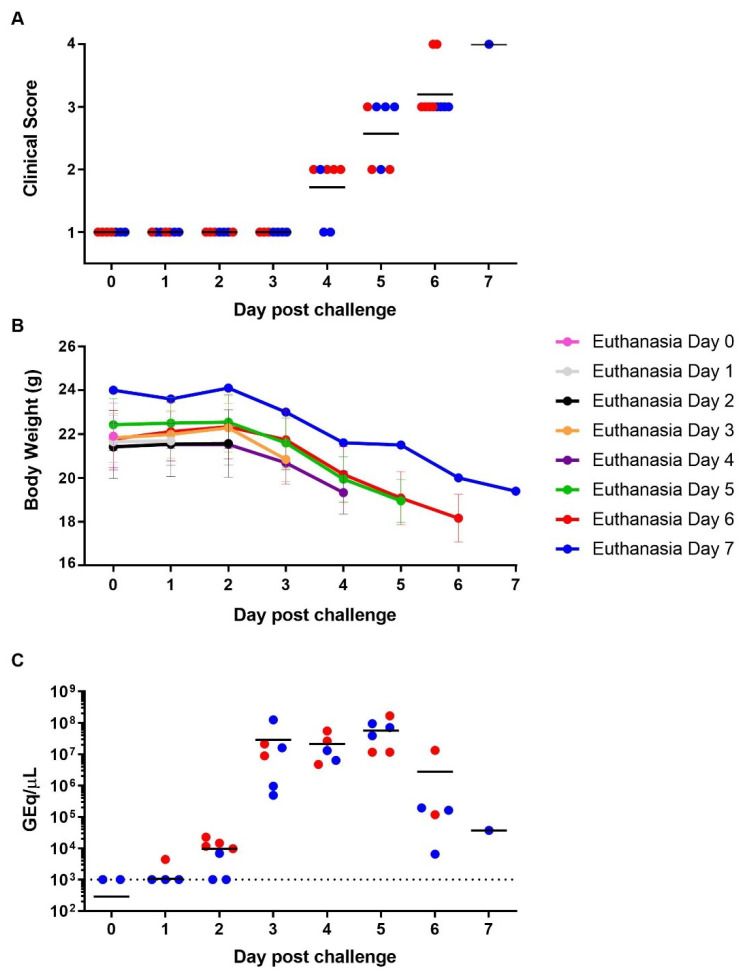
Clinical Scoring, Weight Loss, and Viremia following SUDV Challenge in STAT-1 KO Mice. Individual clinical scores (**A**) and mean body weights grouped by euthanasia day (**B**) were recorded over time. The levels of viral RNA in serum were measured at the time of euthanasia and are presented in genome equivalents per µL (GEq/µL; **C**). Symbols (● and ●) represent individual values from a female or male mouse, respectively. The dotted line indicates the limit of detection of the assay (1 × 10^3^ GEq/μL or approximately 6 pfu/mL). Horizontal and vertical bars indicate means and standard errors, respectively.

Next, we performed a natural history of SUDV-induced pathology study. On day 0, seven mice were euthanized as naïve controls and 46 others were infected with a lethal dose of 1.0 × 10^2^ pfu per animal by i.p. route. Seven pre-selected subjects were scheduled to be euthanized daily through day 6 post-infection. Three additional mice had to be euthanized on day 6 and another one on day 7, due to reaching humane endpoint criteria. Consistent with our previous study, loss of body weight was detectable starting day 3 post-infection, while clinical scores started to increase at day 4 (Figure 3A,B). Both continued to decrease or increase, respectively, through to the end of the study. Virus genome in sera was detected in one animal at day 1 (Figure 3C), followed by five mice at day 2 (average of 9.6 × 10^3^ GEq/µL), and in all mice euthanized thereafter. The maximum amount of viral RNA was detected in sera of subjects euthanized between day 3 and 5, ranging from 2.9 to 5.7 × 10^7^ GEq/µL (correlating to 1.7 and 3.3 × 10^5^ pfu/ml), and concomitant with onset of clinical manifestations and body weight loss (Figure 3A–C). Viral RNA loads in sera decreased, on average, by about 1 log_10_ at day 6 and by 3 log_10_ by day 7.

**Figure 4:**
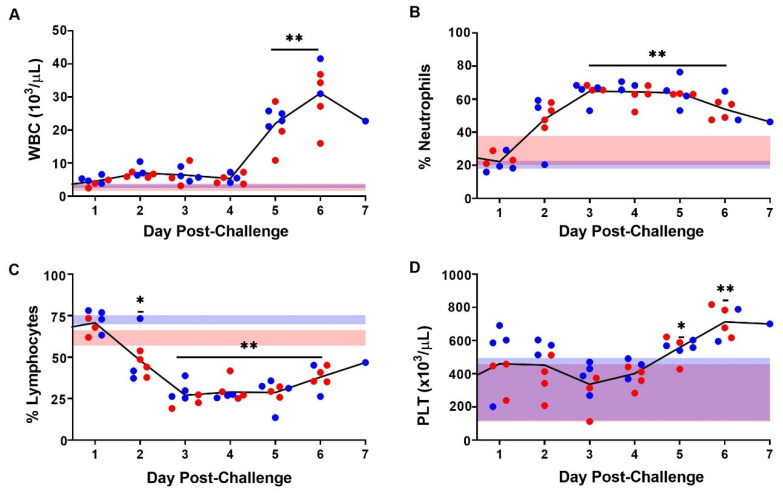
Hematological Changes following SUDV Challenge in STAT-1 KO Mice. Black lines represent average white blood cell count (WBC) (**A**), percent of neutrophils (**B**) percent of lymphocytes (**C**), and platelet counts (PLT) (**D**). Symbols (● and ●) represent individual values from a female or male mouse, respectively. Single (*) and double asterisks (**) indicate statistical differences of *p* < 0.05 and *p* < 0.001, respectively, as compared to mice processed on day 0.

Hematological changes were monitored over the course of infection. Following challenge, total white blood cell counts increased beginning on day 5 (*p* < 0.001, Tukey–Kramer). This trend was present in the percentage of circulating neutrophils by day 3 (*p* > 0.001, Tukey–Kramer; Figure 4A,B), concomitant with the observed increase in viral RNA (Figure 3C) and decrease in the percentage of circulating lymphocytes (Figure 4C). The number of platelets remained relatively unchanged (with substantial animal-to-animal variability) until day 5, at which point an upward trend proceeded until the end of the study (Figure 4D).

**Figure 5:**
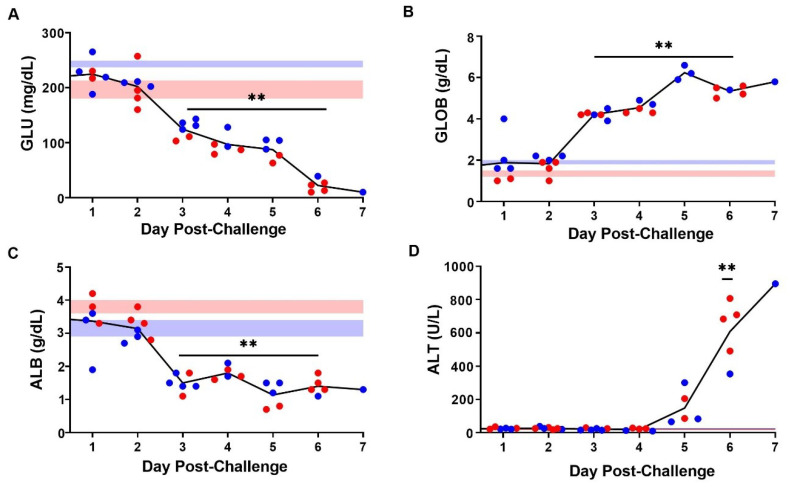
Changes in Serum Chemistry following SUDV Challenge. Black lines represent average glucose (GLU; **A**), globulin (GLOB; **B**), albumin (ALB; **C**), and alanine aminotransferase (ALT; **D**) levels. Symbols (● and ●) represent individual values from a female or male mouse, respectively. Double asterisks (**) indicate significant differences (*p* < 0.001) as compared to uninfected mice processed on day 0.

Among a panel of 14 clinical chemistry parameters measured in whole blood, only four showed a substantial change over the course of the study. Specifically, glucose levels decreased, beginning on day 3 (*p* < 0.001, Tukey-Kramer, Figure 5A), suggestive of hypoglycemia in line with the start of body weight loss (Figure 3B). In parallel, an increase in average globulin levels and a decrease in albumin levels were observed from day 3 onwards (*p* < 0.001 Tukey–Kramer, Figure 5B,C). A steep increase in ALT was observed beginning 5 days post-challenge, with a significant increase measured on day 6 post-infection (*p* < 0.001, Tukey–Kramer, Figure 5D). Altogether, the inversion of globulin and albumin levels and the increase in ALT are suggestive of progressive liver damage.

**Figure 6:**
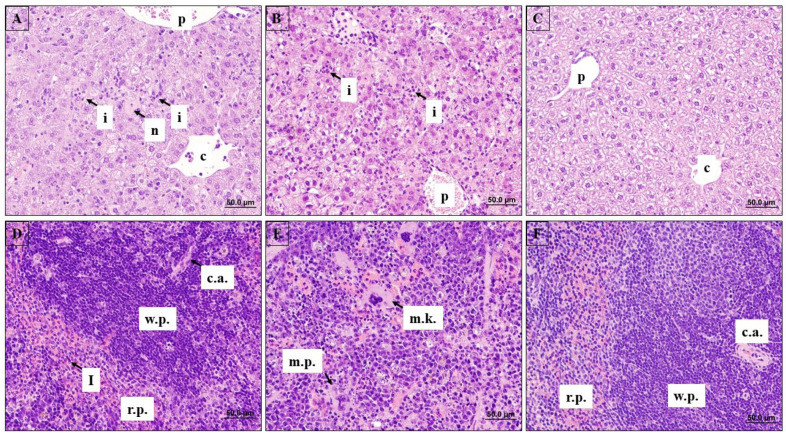
Histopathological Analysis of Liver and Spleen following SUDV Challenge in STAT-1 KO Mice. Representative liver sections showing infiltration of neutrophils (i) and necrosis (n) (**A**, **B**). Liver section from mock-infected animal (**C**). Representative spleen sections showing infiltration of neutrophils (i), megakaryocytes (m.k.), and myeloid precursors (m.p.) (**D, E**). Spleen section from mock-infected animal (**F**). Additional legends: portal vein (p), central vein (c), red pulp (r.p.), white pulp (w.p.), central artery (c.a.).

The liver and spleen of each subject were harvested throughout the study. These organs typically sustain the most severe filovirus-induced damage and are associated with high levels of virus replication. Histological changes were seen in the spleen and liver starting on days 2 and 5, respectively. Noted changes were predominantly characterized by neutrophil inflammation/sinusoidal neutrophilia (Figure 6), with most other changes likely to be secondary. Tissue damage tended to increase in overall severity with each successive day with some minor qualitative changes in certain features as the lesions matured. No meaningful differences were noted in the severity or characteristics of the findings between males and females.

In the liver, focal mixed cell inflammation of the hepatic parenchyma was present in all animals at day 5. Foci were randomly located, variably sized, and largely composed of neutrophils (Figure 6A–C), and occasionally encapsulated by mononuclear cells in larger microabscess formations. The inflammatory foci were often associated with necrosis of single or small clusters of hepatocytes, which sometimes contained eosinophilic cytoplasmic inclusion bodies, suggestive of viral inclusions, but frank necrosis was a relatively minor feature in most animals. More diffuse sinusoidal neutrophilia was also discernible in most animals at day 5, although the severity of this tended to wane from day 6 onward, likely due to consumption of neutrophils and/or obliteration of discernible sinusoids by enlarged, vacuolated hepatocytes. Minimal to severe vacuolation of hepatocytes occurred from day 5, with increasing severity on day 6, which may in part reflect fatty vacuolation associated with reduced feed intake. The various other secondary changes were notably minimal to mild myelopoietic foci composed of large early myeloid precursor cells, minimal focal clumps of eosinophilic fibrillar material, and brown pigment deposition within hepatocytes or Kupffer cells.

In the spleen, neutrophilia of the red pulp (Figure 6D–F) was noted starting on day 2. This increased in severity by day 5 and became less prominent thereafter, likely due to consumption at sites of inflammation. Correlating with this, areas of increased myelopoiesis composed of large, early-stage myeloid-lineage hematopoietic cells (Figure 6E) were consistently present from day 5 onward. Necrotic cell debris, free and within macrophages, was scattered through both the red and white pulp from day 5 onward, and likely consisted of the remains of defunct neutrophils and lymphocytes. Decreased lymphocytes were noted in the splenic white pulp from day 3 onwards, and splenic lymphoid tissue was almost entirely absent from all animals by day 6. Increased brown pigment within splenic macrophages was also noted in most animals beginning day 4 and may represent hemosiderin or bilirubin originating from internal hemorrhage.

### 3.4. Antiviral Treatment of SUDV-Challenged STAT-1 KO Mice

**Figure 7:**
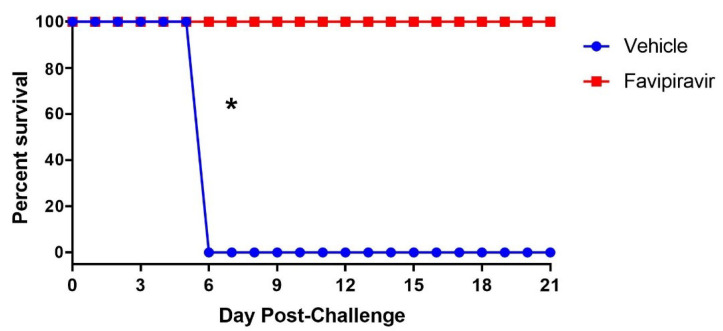
Efficacy of Favipiravir against SUDV in STAT-1 KO Mice. Mice were challenged with 1.0 × 10^2^ pfu of SUDV then treated with 150 mg/kg of favipiravir twice daily for 8 days via oral gavage. The asterisk (*) indicates statistical significance (*p* = 0.002, Fisher’s exact test).

To determine if STAT-1 KO mice could be used to evaluate the efficacy of antivirals, we challenged mice with 1.0 × 10^2^ pfu of SUDV and treated with 150 mg/kg of favipiravir twice daily via oral gavage for a total of 8 days, starting one hour post-challenge. All vehicle control animals exhibited signs of disease starting on day 4 and met euthanasia criteria by day 6 (Figure 7). This was consistent with our results from the SUDV virulence study at an equivalent infective dose. Conversely, treatment with favipiravir resulted in full protection of SUDV-infected mice. Indeed, none of these animals developed clinical signs of disease and only lost a maximum average of 4% body weight at any given day of the study.

## 4. Discussion

Although a significant amount of work has been conducted in filovirus disease in small-rodent models [50], data on the characterization of virulence and pathogenesis of wild-type viruses other than Ebola virus or Marburg virus in immunocompromised mouse models are still sparse. Yet, these data are critical for determining which relevant rodent animal model (s) may be used to achieve reproducible endpoints following infection, in order to get comparable virulence and pathogenesis data. The use of a consensus immunocompromised mouse model of filovirus disease will also be beneficial in screening for filovirus countermeasures.

Here, the susceptibility of the STAT-1 KO mouse model to five wild-type filovirus species pathogenic in humans was initially tested. The IP route of infection was chosen in our study as it has been extensively used in previous studies characterizing small animal models of filovirus disease to study the pathogenicity and screening of antivirals and vaccines. Moreover, while we acknowledge that using another route could result in an altered virulence for each virus tested, some studies have reported similar disease outcomes and timeline whether IP, intranasal, or aerosol were used, at equivalent doses [48,51].

All virus stocks came from the same repository, with the advantage that they were well characterized, properly stored, free of contaminants, and commercially available, thereby ensuring reliable results between studies and research groups over time. Although some virus strains were different, our data were consistent with Raymond et al. (2011) who previously used the same mouse model with comparable infective doses. In the previous study, SUDV (strain Boniface), EBOV (strain Zaire Kikwit), and MARV (strain Musoke) caused clinical manifestations of disease starting on day 3, 5, or 5, respectively, with weight loss onset occurring 3–4 days post-challenge and with fatality within 7 days [52]. In the present study, animals developed clinical signs of disease and started losing weight at day 3 or 4 following SUDV (strain Gulu), EBOV (strain Zaire Kikwit), MARV (strain Angola), and BDBV infection. Mortality also occurred within a 7-day window, with virulence, ranking from the most least virulent filovirus species, as follows: SUDV (100%), MARV (80%), EBOV (40%), BDBV (20%), and Tai Forest virus (no mortality). In previous studies using IFNAGR [39] and IFNAR [43] KO mice on a C57BL/6J background, different observations were made. Specifically, at comparable infective doses, EBOV strain Zaire Kikwit was more virulent than SUDV strain Gulu, 100% and 60% mortality, respectively) in IFNAGR [39]. Likewise, EBOV was more virulent than SUDV strain Boniface (13% and 8% mortality, respectively), and SUDV strain Gulu (no mortality) in IFNAR [43] mice. Furthermore, no animals succumbed to disease following BDBV challenge. Provided that there is no variability in the ability to antagonize interferon response between pathogenic ebolaviruses, changes in virulence could be explained by the fact that disrupting STAT-1 expression is more efficient at blocking type I or II IFN response than in IFNAGR and IFNAR KO mice, as STAT-1 signals the binding of both IFN types to cell-surface receptors [53]. Differences in genetic traits between 129 and C57BL/6J mouse backgrounds could also be involved in the outcome of disease following filovirus challenge, as infection with high doses was shown to increase survival of the latter mouse strain [23] and virulence was found to vary greatly based on the different C57BL/6J mouse strains used [23].

TAFV and BDBV pathogenesis has been less investigated compared to other filoviruses. Only one case of TAFV infection was reported in 1994 in Western Côte d’Ivoire [2], where the patient developed a temporary febrile disease following necropsy of a chimpanzee whose colony had been severely affected by episodes of a hemorrhagic fever-like disease (25% mortality) [54]. Conversely, BDBV was responsible for two outbreaks involving more than 100 cases each in 2007 and 2012, and caused about 30% mortality [2]. Difference in virulence between both species was also reported in a humanized mouse model, as TAFV, BDBV, SUDV, and EBOV caused 18, 29, 71, and 93% mortality, respectively [55]. TAFV RNA in blood was also significantly lower than that observed for all the other ebolaviruses. However, it is interesting to note that neither TAFV nor BDBV caused disease in IFNAR and IFNAGR KO mice, while SUDV caused 40 to 60% mortality [39,43].

SUDV (strain Gulu and Boniface)-infected NHPs rapidly become viremic and develop thrombocytopenia, lymphopenia, blood coagulation disorders; increases in liver enzyme activities, including ALT and ALP; and accumulation of serum byproducts of protein metabolism, including BUN and CRE [56,57,58,59,60]. Alterations of some of these hematology and blood chemistry parameters were also found in guinea pigs [27], ferrets [61,62], IFNAR [43] and IFNAGR [39] mice on a C57BL/6J background, and in our study with STAT-1 KO mice. Consistent with previous observations in EBOV-infected IFNAGR KO mice [39] and human patients [63], we observed neutrophilia following SUDV challenge in our STAT-1 KO mice. Histopathology findings indicated that SUDV replicated in the liver and spleen of mice in our study, similar to NHPs [56,57,58,59,60], guinea pigs [27,64], and IFNAR KO mice [43], with features somewhat consistent with those described in the corresponding organs of EBOV-infected patients [65]. Interestingly, lack of STAT-1 and IFNAGR expression have the same effect on disrupting type I and II interferon signaling pathways. However, there were pathological features in STAT-1 KO mice that differed from the IFNAGR KO mouse model [39]. These included greater intensity and homogeneity of the neutrophil inflammation, presence of hepatocellular vacuolation, longer onset time of the hepatic lesion, absence of focal coagulative necrosis and thrombi within the liver, absence of cystic dilatation within the spleen, and absence of discernible splenic histiocytosis. Altogether, our data suggest that the STAT-1 KO mouse model on a 129 background is relevant for studying wild-type SUDV disease, as an alternative to using larger models.

Favipiravir has been shown to be effective against wild-type EBOV in immunosuppressed mouse models [28,29,30,39], mouse-adapted EBOV and MARV in immunocompetent mouse models [29,66,67], adapted SUDV in a guinea pig model [64], and wild-type EBOV or MARV in NHPs [68,69]. In the present study, we demonstrate that favipiravir was fully protective in SUDV-challenged STAT-1 KO mice, suggesting that this model may be used as a screen for testing antivirals against SUDV. Future studies might focus on determining the minimal protective dose. It is interesting to note, however, that this model has previously been reported as inappropriate for testing VSV-based vaccines [42].

In conclusion, the STAT-1 KO (129S background) mouse model displayed distinct susceptibility levels to five wild-type filovirus species and shared some of the hematology/blood chemistry changes and histopathological lesions seen in larger but less cost-effective models for wild-type SUDV infection, including ferrets and NHPs. Since STAT-1 KO mice are overly susceptible to wild-type filoviruses by virtue of not being able to respond to type II IFN, this model could be an interesting standard tool to test the virulence of new isolates, in order to compare with other known filoviruses, and for screening candidate vaccines and therapeutics. This model will also be useful in identifying virulence factors by doing side-by-side comparison studies using other immunocompromised mouse models, such as IFNAR, on the same 129 background.

## Data Availability

The data presented in this study are available on request from the corresponding authors.
